# Dissecting the association between gut microbiota and liver cancer in European and East Asian populations using Mendelian randomization analysis

**DOI:** 10.3389/fmicb.2023.1255650

**Published:** 2023-09-18

**Authors:** Hua Jiang, Tianjun Song, Zhongyi Li, Lingxuan An, Chiyi He, Kai Zheng

**Affiliations:** ^1^Department of Gastroenterology, The First Affiliated Hospital of Wannan Medical College, Wuhu City, China; ^2^Department of Medicine II, University Hospital, Munich, Germany; ^3^Department of General, Visceral, Transplant, Vascular and Thoracic Surgery, Ludwig-Maximilians-University of Munich, Munich, Germany; ^4^Department of Trauma Microsurgery, Zhengzhou Central Hospital Affiliated to Zhengzhou University, Zhengzhou, China

**Keywords:** gut microbiota, liver cancer, Mendelian randomization, Europeans, East Asians

## Abstract

**Background:**

Ample evidence suggests an important role of the gut microbiome in liver cancer, but the causal relationship between gut microbiome and liver cancer is unclear. This study employed Mendelian randomization (MR) analysis to examine the causal relationship between the gut microbiome and liver cancer in European and East Asian populations.

**Methods:**

We sourced genetic variants linked to gut microbiota from the MiBioGen consortium meta-analysis, and procured liver cancer genome-wide association study (GWAS) summary data from the FinnGen consortium and Biobank Japan. We employed the inverse variance weighted method for primary statistical analysis, fortified by several sensitivity analyses such as MR-PRESSO, MR-Egger regression, weighted median, weighted mode, and maximum likelihood methods for rigorous results. We also evaluated heterogeneity and horizontal pleiotropy.

**Results:**

The study examined an extensive set of gut microbiota, including 131 genera, 35 families, 20 orders, 16 classes, and 9 phyla. In Europeans, ten gut microbiota types displayed a suggestive association with liver cancer (*p* < 0.05). Notably, *Oscillospira* and *Mollicutes RF9* exhibited a statistically significant positive association with liver cancer risk, with odds ratios (OR) of 2.59 (95% CI 1.36–4.95) and 2.03 (95% CI 1.21–3.40), respectively, after adjusting for multiple testing. In East Asians, while six microbial types demonstrated suggestive associations with liver cancer, only *Oscillibacter* displayed a statistically significant positive association (OR = 1.56, 95% CI 1.11–2.19) with an FDR < 0.05. Sensitivity analyses reinforced these findings despite variations in *p*-values.

**Conclusion:**

This study provides evidence for a causal relationship between specific gut microbiota and liver cancer, enhancing the understanding of the role of the gut microbiome in liver cancer and may offer new avenues for preventive and therapeutic strategies.

## Introduction

Liver cancer is a global public health concern. Ranked as the sixth most common cancer and the third leading cause of cancer-related deaths worldwide (Sung et al., 2021), liver cancer has a particularly high incidence in East Asia and a growing prevalence in European countries (Liu et al., 2019). While several risk factors for liver cancer have been identified (McGlynn et al., 2021), such as chronic hepatitis B and C infections, alcohol consumption, and non-alcoholic fatty liver disease, the multifactorial nature of the disease suggests that additional, hitherto unrecognized factors may contribute to its pathogenesis. Among these potential factors, the role of the gut microbiome is attracting increasing attention (Yu and Schwabe, 2017; Ma et al., 2018; Zhang et al., 2021).

The gut microbiome, the community of microorganisms residing in the human gastrointestinal tract, has been implicated in the development and progression of numerous diseases, including various types of cancer (Gopalakrishnan et al., 2018; Tong et al., 2021). A growing body of evidence suggests that the composition and function of the gut microbiome can influence the development of liver diseases, such as liver cirrhosis and non-alcoholic fatty liver disease (Wang et al., 2021; Tilg et al., 2022). Furthermore, several studies have indicated that gut microbial dysbiosis, characterized by an imbalance in the microbial community, may be involved in the development and progression of liver cancer (Yu and Schwabe, 2017; Ma et al., 2018). These findings have provided impetus for research into the intricate relationship between the gut microbiome and liver cancer.

However, establishing a causal link between gut microbiome and liver cancer has been challenging due to confounding factors and reverse causation. Mendelian randomization (MR) offers a robust analytical tool to address these challenges (Davey Smith and Hemani, 2014; Zuber et al., 2022), as it exploits genetic variants as instrumental variables to assess the causal effect of an exposure (in this case, the gut microbiome) on an outcome (liver cancer). This approach can be particularly useful in understanding the genetic and environmental interactions in the pathogenesis of liver cancer among different populations, such as Europeans and East Asians.

In this article, we aim to utilize MR analysis to explore the association between gut microbiome and liver cancer in European and East Asian populations. By focusing on these two populations that have contrasting incidences of liver cancer and distinct gut microbial profiles, we seek to gain novel insights into the potential role of the gut microbiome in liver cancer development and identify possible avenues for preventive and therapeutic interventions.

## Methods

**Figure 1:**
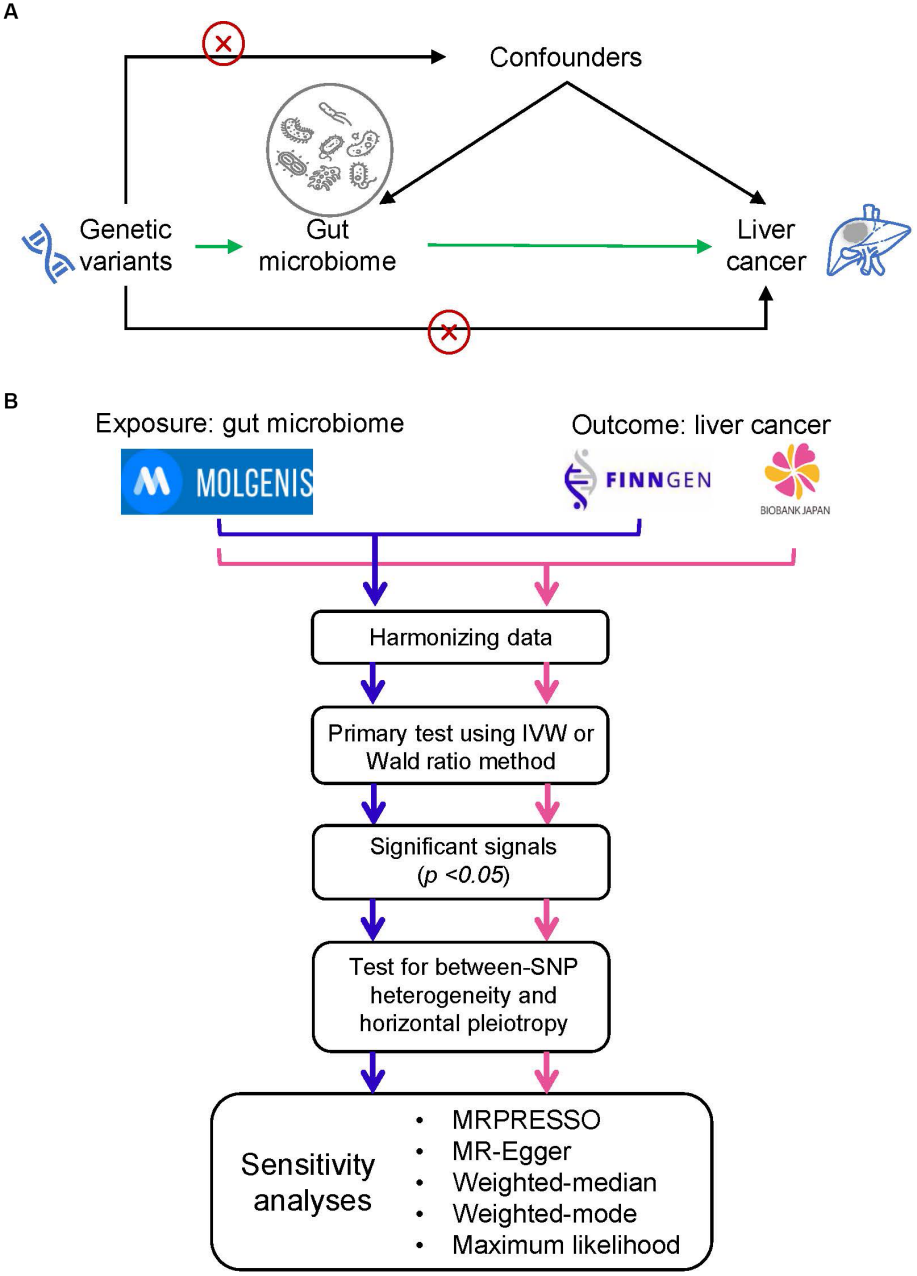
Study design and flowchart. **(A)** The basic schema of Mendelian randomization (MR) analysis, in which we set gut microbiota as the exposure and the liver cancer as the outcome. The cross signs are used to indicate the Mendelian randomization assumptions that (i) instrumental variables (IVs) did not affect the confounders between exposure and outcome and (ii) IVs did not affect the outcome through any other pathway. **(B)** Data analysis process. We performed two independent MR analyses with the same exposure data but different outcome data (i.e., liver cancer data from FinnGEN and Biobank Japan). We firstly conducted a primary screening to identify putative significant microbe signals and then performed a set of MR analysis to ensure the primary findings. MOLGENIS refers to a modular web application for scientific data including the GWAS summary data of gut microbiota.

The study schema of this study was shown in Figure 1A. We defined the gut microbiota as the exposure and liver cancer as the outcome. Genetic variants that significantly associated with gut microbiota were employed as instrumental variables (IVs). In the framework of MR study, the IVs have to meet the following three criteria: (1) IVs were significantly associated with the exposure; (2) IVs did not affect the confounders between exposure and outcome; and (3) IVs did not affect the outcome through any other pathway (Burgess and Thompson, 2017).

### Sources of genome-wide association study summary data

Genetic variants associated with gut microbiota were retrieved from the most extensive meta-analysis of genome-wide studies concerning gut microbiota composition to date, carried out by the MiBioGen consortium (Kurilshikov et al., 2021). This investigation encompassed 18,340 participants from 24 different groups, with the majority being of European descent (*n* = 13,266). It focused on the variable regions V4, V3–V4, and V1–V2 of the 16S rRNA gene in order to characterize the microbial make-up and to perform taxonomic categorization through direct taxonomic binning. The study employed microbiota quantitative trait loci (mbQTL) mapping to pinpoint host genetic variants that corresponded to genetic sites linked to the varying abundance levels of bacterial species within the gut microbiota. Within this study, the genus was the most specific level of taxonomy investigated, and 131 genera with an average abundance exceeding 1% were discovered, among which 12 genera were previously unknown (Kurilshikov et al., 2021). Moreover, the study encompassed nine phyla, 16 classes, 20 orders, and 35 families (Kurilshikov et al., 2021).

The GWAS summary data of liver cancer of Europeans and East Asians were retrieved from FinnGen consortium R7 release data (Mitja et al., 2022) and Biobank Japan (Nagai et al., 2017), respectively. In the FinnGen study, a total of 518 liver cancer cases and 308,636 controls were included in the GWAS. In Biobank Japan, 2,122 cases and 159,201 controls were included in the GWAS.

### Instrumental variables

The selection of IVs adhered to these criteria: (1) Single nucleotide polymorphisms (SNPs) that were linked to each microbiota unit and met the locus-wide significance threshold (*p* < 1.0 × 10^−5^) were earmarked as potential IVs (Sanna et al., 2019; Li et al., 2022); (2) The 1,000 Genomes project’s European sample data served as the reference panel for computing the linkage disequilibrium (LD) among SNPs. Of these, only the SNPs with the lowest *p*-values were kept if they had an *R*^2^ value below 0.01 and were within a clumping window size of 10,000 kb; (3) SNPs that had a minor allele frequency (MAF) of 0.01 or less were excluded; (4) In cases where palindromic SNPs were present, the forward strand alleles were inferred using allele frequency data. The potency of IVs was evaluated by determining the F-statistic using the equation *F = R^2^ × (N − 1 − K) / ((1 − R^2^) × K)*. Here, R^2^ signifies the portion of the exposure’s variance elucidated by the genetic variants, *N* stands for the sample size, and *K* indicates the quantity of instruments (Li et al., 2022). An F-statistic exceeding 10 implied the absence of any substantial weak instrumental bias.

### Statistical analysis

The statistical flow chart was shown in Figure 1B. After harmonizing data of exposure and outcome, we performed a primary screening test using inverse variance weighted (IVW) method or Wald ratio method to identify the significant microbe signals. For microbe that reached the traditional significance threshold (*p* value <0.05), we performed a set of additional analyses to ensure the robustness of primary findings. Firstly, we performed tests for horizontal pleiotropy through the application of the MR-PRESSO global test (Verbanck et al., 2018), and outliers, specifically SNPs with a *p*-value less than 0.05, were eliminated if the presence of horizontal pleiotropy was confirmed. Secondly, we assessed between-SNP heterogeneity by implementing the IVW method, which was based on the SNPs left post-pleiotropy adjustment. The Cochran’s Q statistic was utilized to ascertain the existence of heterogeneity, and SNPs with a *p*-value higher than 1.00 in the MR-PRESSO analysis were discarded if significant heterogeneity was identified (*p*-value of Cochran’s Q statistic being less than 0.05). Moreover, we executed a range of sensitivity analyses employing five alternative methodologies: MRPRESSO, MR-Egger regression, weighted median, weighted mode, and maximum likelihood (ML) methods. MR-Egger regression is constructed on the InSIDE presumption (INstrument Strength Independent of Direct Effect) and is comprised of three parts: (i) a test for directional pleiotropy, (ii) a test to identify a causal effect, and (iii) a calculation of the causal effect (Burgess and Thompson, 2017). The weighted median and weighted mode methods serve as robust strategies when more than half of the SNPs are deemed invalid instruments (Hartwig et al., 2017). The ML approach is akin to the IVW method, premised on the assumptions that heterogeneity and horizontal pleiotropy are absent. If these underlying presumptions hold true, the outcomes will not display any bias, and the standard errors generated will be comparatively smaller than those from the IVW method (Pierce and Burgess, 2013). Furthermore, we carried out a “leave-one-out” examination to detect influential SNPs by omitting each instrumental SNP in turn. We estimated the statistical power of the MR analysis with the assistance of the mRnd website (Burgess, 2014). False-discovery-rate (FDR) was applied to adjust for multiple testing.

## Results

**Figure 2:**
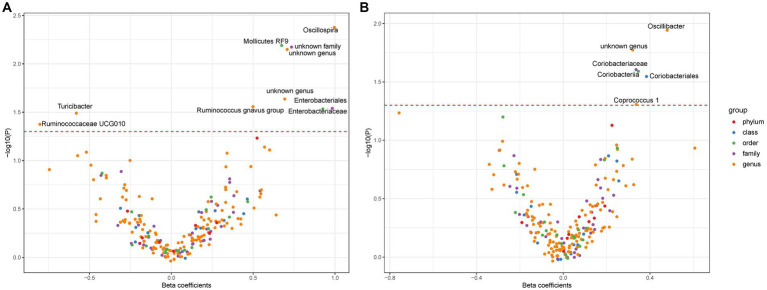
The association between gut microbiota and liver cancer. Subfigure **(A,B)** shows results in Europeans and East Asians, respectively. The red dashed line denotes statistical significance threshold (i.e., *p* < 0.05). The points were jittered to avoid overlap.

In this investigation, an extensive set of gut microbiota was analyzed, encompassing 131 genera, 35 families, 20 orders, 16 classes, and 9 phyla, to perform an initial screening test. Among Europeans, we identified ten types of gut microbiota demonstrating an association with liver cancer, utilizing a significance threshold of *p* < 0.05. This includes six genera (namely, *Oscillospira*, *Mediterraneibacter gnavus* group, *Turicibacter*, *Ruminococcaceae UCG010*, and two unidentified genera), two families (*Enterobacteriaceae* and one unidentified family), and two orders (*Enterobacteriales* and *Mollicutes RF9*) (Figure 2A). It is noteworthy that *Enterobacteriaceae* and *Enterobacteriales* were represented by the same IVs. In East Asians, six microbial types exhibited significant associations with liver cancer, comprising three genera (*Oscillibacter*, *Coprococcus* 1, and an unidentified genus) and *Coriobacteriaceae*, *Coriobacteriia*, and *Coriobacteriales*, which utilized identical IVs (refer to Figure 2B). Consequently, we proceeded with four bacterial genera, *Enterobacteriaceae*, and *Mollicutes RF9* for Europeans, and two bacterial genera along with *Coriobacteriaceae* for East Asians, in further analyses (Table 1).

**Table 1:** Mendelian randomization analysis statistics.

Populations	Gut microbe	No. of IV	F-statistics	Between-SNP heterogeneity		Horizontal pleiotropy		Statistical power to detect OR < 0.9 or > 1.1 (%)	Statistical power to detect OR between 0.9 and 1.1 (%)
				Q-value	*p* value	Egger-intercept	*p* value		
Europeans	*Oscillospira*	8	91.8	2.628	0.917	−0.074	0.600	84	65
	*Ruminococcaceae UCG010*	6	43.2	3.635	0.603	−0.028	0.738	75	48
	*Mediterraneibacter gnavus group*	12	114.8	12.353	0.338	0.029	0.808	95	74
	*Turicibacter*	9	74.6	6.058	0.641	0.054	0.670	81	53
	*Enterobacteriaceae*	7	74.3	5.858	0.439	−0.114	0.587	83	60
	*Mollicutes RF9*	13	174.8	6.044	0.914	−0.0004	0.994	100	89
East Asians	*Oscillibacter*	7	65.3	5.655	0.463	−0.033	0.763	81	62
	*Coprococcus 1*	9	101.1	7.431	0.491	0.024	0.684	93	72
	*Coriobacteriaceae*	15	122.3	12.644	0.555	0.017	0.703	100	86

For the selected gut microbes, between 6 and 15 IVs were employed (see Supplementary Tables S1–S9), with mean F-statistics ranging from 43.2 to 174.8 (Table 1). We did not observe heterogeneity between SNPs or horizontal pleiotropy for any of the gut microbes (Table 1). The IVs provided sufficient statistical power, ranging from 83 to 100%, to detect an odds ratio (OR) below 0.9 or above 1.1. However, the statistical power diminished to varying extents when attempting to discern an OR between 0.9 and 1.1 (Table 1).

**Figure 3:**
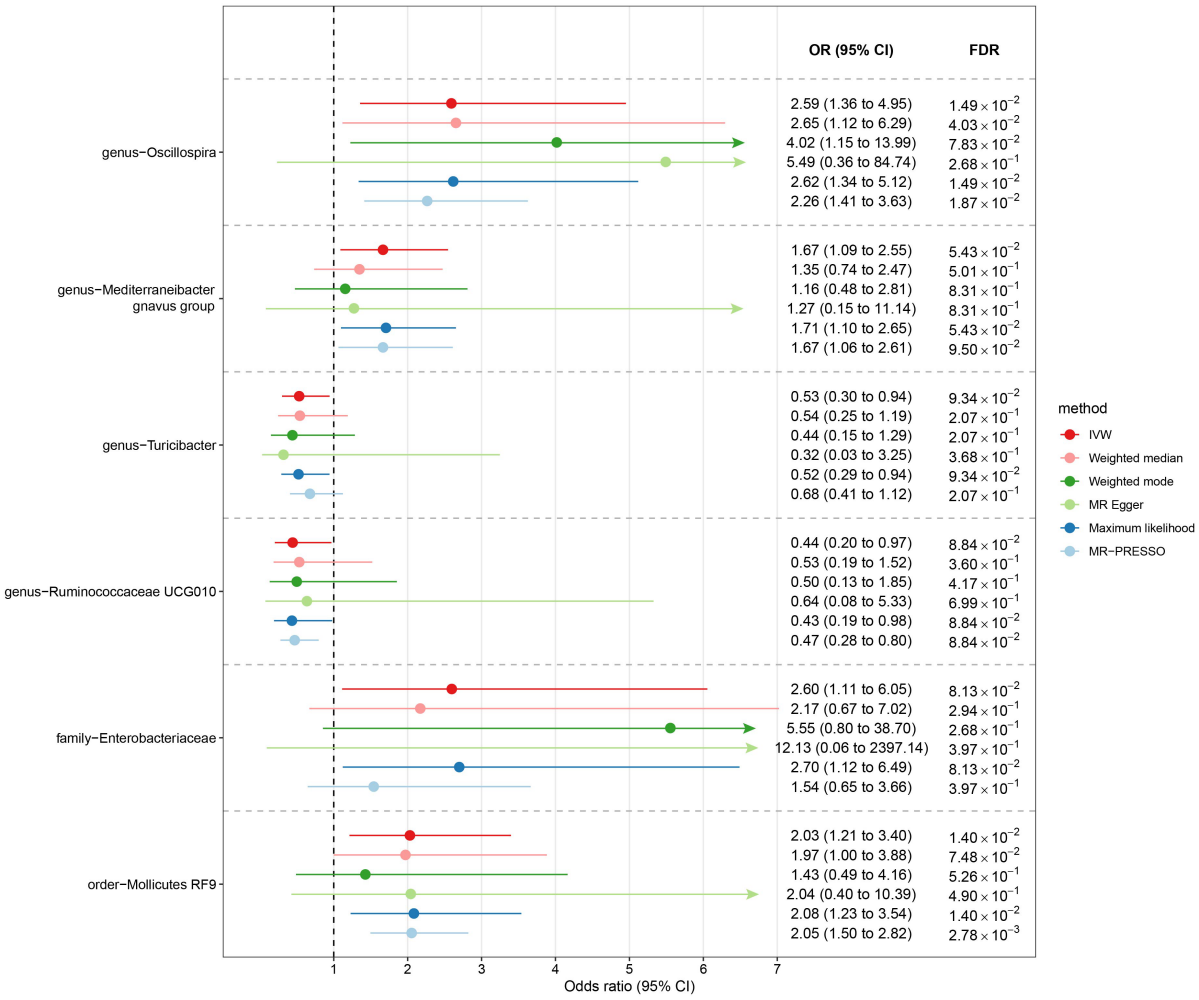
The association between gut microbiota and liver cancer in Europeans. Lines in the figures denote the 95% confidence interval (CI) of the odds ratio and arrows in the figures are used to indicate that the lower or upper bound of the 95% CI was beyond the range of the x-axis. “FDR” stands for “False Discovery Rate” and “IVW” refers to “Inverse Variance Weighted”.

**Figure 4:**
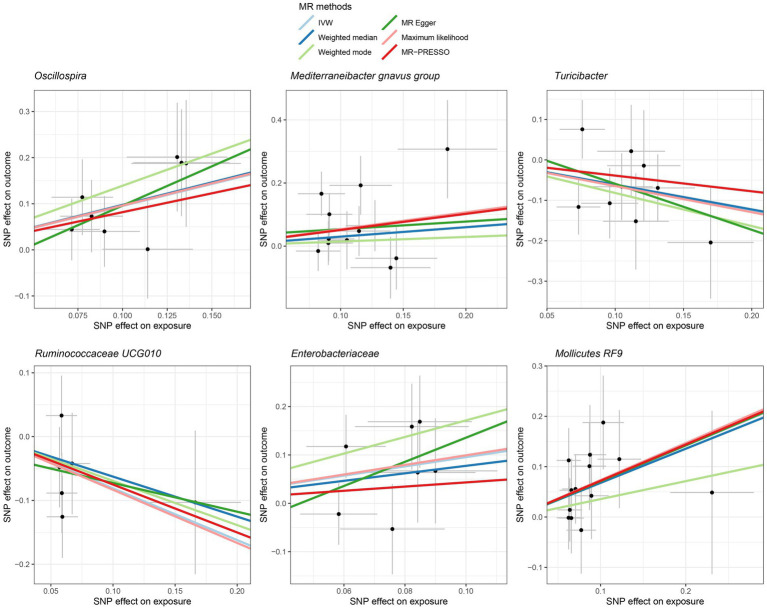
Scatter plot showing the SNP effects on both gut microbiota and liver cancer in Europeans. The gray error bars denote the 95% confidence intervals of the effects.

Among Europeans, a significantly positive association between *Oscillospira* and liver cancer risk was observed, exhibiting an OR of 2.59 (95% CI 1.36–4.95; FDR = 1.49 × 10^−2^) (Figure 3). This association was corroborated through alternative MR methodologies, including weighted-median (OR = 2.65, 95% CI 1.12–6.29; FDR = 4.03 × 10^−2^), ML (OR = 2.62, 95% CI 1.34–5.12; FDR = 1.49 × 10^−2^), and MRPRESSO (OR = 2.26, 95% CI 1.41–3.63; FDR = 1.87 × 10^−2^). Additionally, the IVW method indicated a significantly positive association between *Mollicutes RF9* and liver cancer, with an OR of 2.03 (95% CI 1.21–3.40; FDR = 1.40 × 10^−2^), which was also validated via ML and MRPRESSO methods (Figure 3). The remaining four types of gut microbiota did not exhibit significant associations post-correction for multiple testing (FDR > 0.05). Despite variability in OR estimates and corresponding FDR values, all six MR methods yielded consistent causal estimates between gut microbes and liver cancer (Figure 4). For *Oscillospira* and *Mollicutes RF9*, no influential outlier was identified via “leave-one-out” analysis (Supplementary Figure S1), further supporting the robustness of our findings.

**Figure 5:**
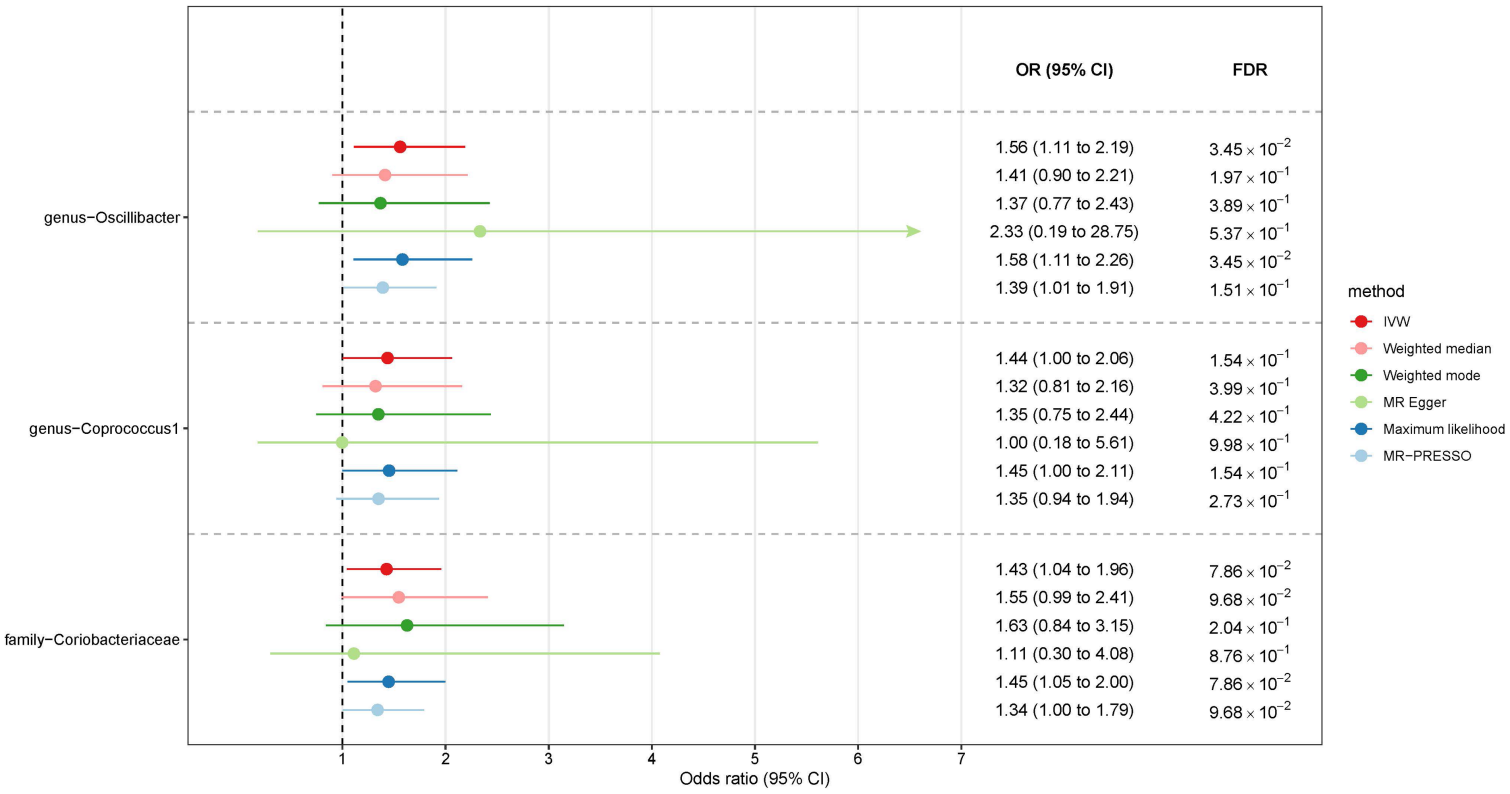
The association between gut microbiota and liver cancer in East Asians. Lines in the figures denote the 95% confidence interval (CI) of the odds ratio and arrows in the figures are used to indicate that the lower or upper bound of the 95% CI was beyond the range of the x-axis. “FDR” stands for “False Discovery Rate” and “IVW” refers to “Inverse Variance Weighted”.

**Figure 6:**
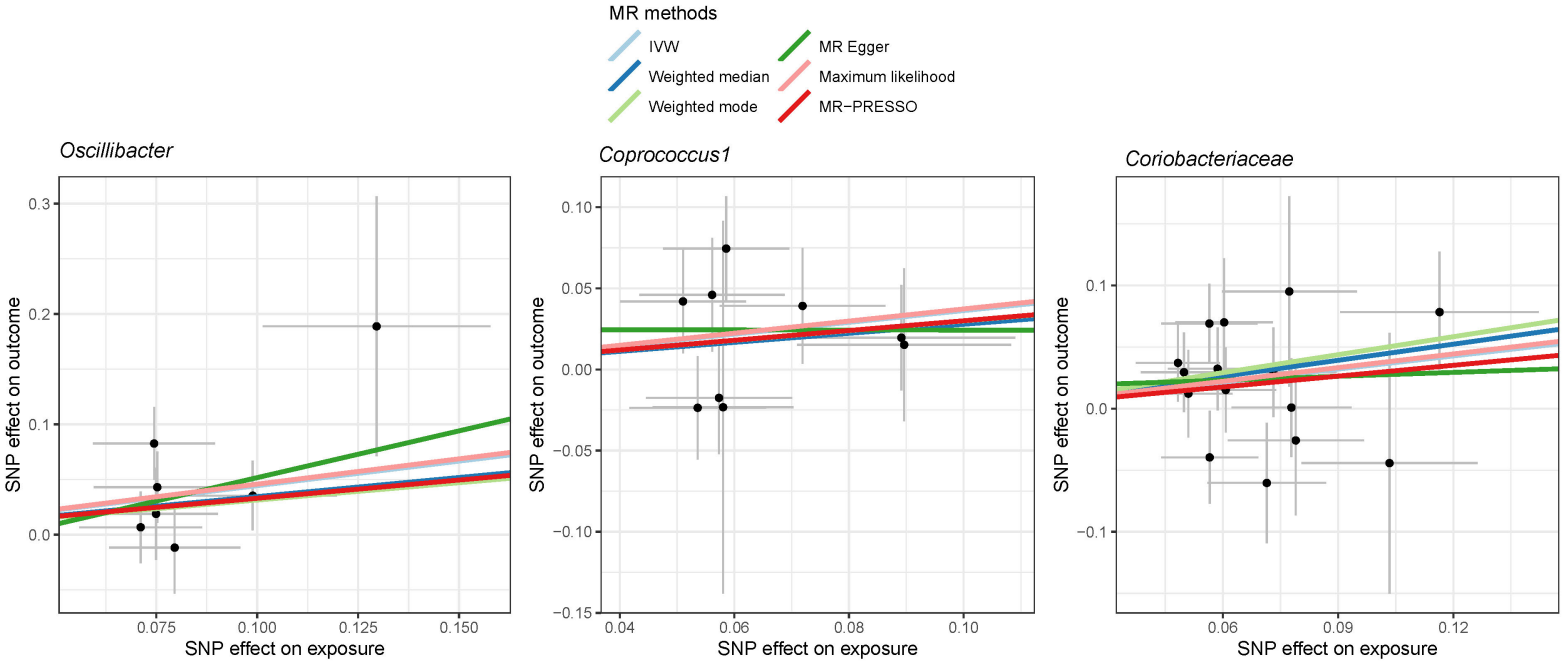
Scatter plot showing the SNP effects on both gut microbiota and liver cancer in East Asians. The gray error bars denote the 95% confidence intervals of the effects.

In the case of East Asians, the IVW method only revealed a statistically significant positive association between *Oscillibacter* and liver cancer, with an OR of 1.56 (95% CI 1.11–2.19; FDR = 3.45 × 10^−2^) after correction for multiple testing (Figure 5). This association was further supported by ML and MRPRESSO methods. For *Coprococcus 1* and *Coriobacteriaceae*, although all MR approaches indicated a positive association with liver cancer, these associations did not meet the statistical significance threshold of FDR < 0.05 (Figures 5, 6). Notably, potential outliers among the IVs for three types of microbiotas were visually apparent in leave-one-out plots (Supplementary Figure S2). However, the MRPRESSO method did not identify any significant outliers (global test *p* > 0.05).

## Discussion

In this study, we embarked on an investigation to unravel the association between gut microbiome and liver cancer in European and East Asian populations, employing the MR analysis. Our results indicate that there is a potential association between specific microbes and liver cancer risk, albeit with variation between the two populations. The gut microbiome, an intricate ecosystem harboring trillions of microorganisms, has been an area of burgeoning research due to its role in human health and disease (Lynch and Pedersen, 2016; Singh et al., 2017). Our study leverages the GWAS summary data from the MiBioGen consortium, which is the largest GWAS for microbiome, and liver cancer data from FinnGen and Biobank Japan, providing a robust foundation for our analyses. Given the global burden of liver cancer and the emerging role of the gut microbiome in health and disease, this research represents a critical step in the continuing efforts to combat liver cancer through a deeper understanding of its multifaceted etiology.

Among Europeans, our findings suggest an association between liver cancer risk and several microbes, including *Oscillospira*, *Mediterraneibacter gnavus group*, and *Turicibacter*. *Oscillospira* has previously been linked with metabolic functions and the breakdown of complex carbohydrates (Lordan et al., 2020; Palmas et al., 2021). *Oscillospira* has been known to be involved in the fermentation of complex polysaccharides and production of short-chain fatty acids (SCFAs) such as butyrate (Berni Canani et al., 2016). SCFAs have been reported to possess anti-inflammatory properties and play a role in maintaining gut barrier integrity (Trompette et al., 2022). A compromised gut barrier could lead to increased translocation of bacterial products into the liver, resulting in chronic inflammation—a known risk factor for liver cancer. Given these characteristics, it might seem counterintuitive to posit *Oscillospira* as a probiotic concerning liver cancer. However, our findings underscore a positive association between *Oscillospira* and liver cancer, suggesting its potential adverse effect on liver health. This observation is aligned with the conclusions drawn by Ponziani et al. (2019a,b), where a higher abundance of *Oscillospira* was reported in the hepatocellular carcinoma (HCC) group compared to controls. Therefore, it becomes imperative to conduct more in-depth and comprehensive research to ascertain the nature and implications of this relationship. Exploring factors such as the overall microbial environment, potential pathogenic strains of *Oscillospira*, or its interactions with other liver-affecting agents may shed light on this complex association.

The *Mediterraneibacter gnavus* group is intriguing as it has been associated with both beneficial and harmful effects (Joossens et al., 2011; Lozano et al., 2022). While some members of this group are involved in the fermentation of dietary fibers and production of butyrate (Nilsen et al., 2020), others have been linked to the production of pro-inflammatory molecules (van Soest et al., 2020). The dual role of *Mediterraneibacter gnavus* in gut health and inflammation could explain its association with liver cancer, as chronic inflammation might promote hepatic carcinogenesis. A study by Behary et al. (2021) showed a significant enrichment of *Mediterraneibacter gnavus* in both the nonalcoholic fatty liver disease (NAFLD)-HCC and NAFLD-cirrhosis groups compared to healthy controls. *Mediterraneibacter gnavus*, previously classified as *Ruminococcus gnavus*, has been moved from the genus *Ruminococcus* in the *Ruminococcaceae* family to its current placement in the genus *Mediterraneibacter* of the *Lachnospiraceae* family (Togo et al., 2018). This distinction between the *Ruminococcaceae* and *Lachnospiraceae* families holds significance, as each family possesses unique metabolic roles and associations with human health (Flint et al., 2012; Donaldson et al., 2016; Rivière et al., 2016). Previous studies have linked *Mediterraneibacter gnavus* with gut dysbiosis (Crost et al., 2023). Such imbalances in the gut microbiome can be implicated in various gastrointestinal disorders, notably inflammatory bowel disease and irritable bowel syndrome (Hall et al., 2017; Chen et al., 2023). Moreover, emerging evidence indicates that these microbial changes, especially with species like *Mediterraneibacter gnavus*, may play a role in the development of liver diseases, including liver cancer, potentially through the gut-liver axis (Ponziani et al., 2019a; Komiyama et al., 2021). This bacterium can produce metabolites that, when translocated to the liver, might exacerbate conditions like NAFLD or even promote the progression to liver cancer (Komiyama et al., 2021). Beyond the gut, the dysbiosis featuring *Mediterraneibacter gnavus* has been implicated in other conditions such as metabolic syndrome and some autoimmune diseases (Grahnemo et al., 2022; Silverman et al., 2022). These associations underscore the importance of a balanced gut microbiome in systemic health. This gut microbe’s role in carcinogenesis is a burgeoning field of research. While direct evidence linking *Mediterraneibacter gnavus* to cancer is still emerging, its role in chronic inflammation – a known risk factor for several cancers – makes it an interesting subject for further studies. The chronic inflammation promoted by gut dysbiosis could potentially create an environment conducive to genetic mutations and tumor growth.

*Turicibacter*, though less studied, has been implicated in modulating host immune responses and gut metabolism (Lynch et al., 2023). Dysregulation in immune response and metabolism could create a microenvironment conducive to cancer development. The interaction of *Turicibacter* with other gut microbes might also influence the overall gut microbiome, which in turn could have systemic effects on liver health. The positive association between Mollicutes RF9 and liver cancer observed in this study could be indicative of a complex interplay between this microbial group and hepatic health. Mollicutes are a class of bacteria known for lacking a cell wall and having a small genome, which suggests a highly specialized and adaptable lifestyle (Trachtenberg, 2005). Their association with liver cancer may stem from their potential role in modulating immune responses, metabolizing dietary components, and interacting with other microbes within the gut (Li et al., 2020). For instance, as Mollicutes lack a cell wall, they may be more invasive and capable of crossing the gut barrier, which could lead to the translocation of bacterial products into the liver. This, in turn, might contribute to chronic inflammation, a well-established risk factor for liver cancer.

In East Asians, *Oscillibacter*, *Coprococcus 1*, and *Coriobacteriaceae* were found to be associated with liver cancer risk. *Oscillibacter* has been implicated in the production of SCFAs (Liu et al., 2022), which are critical for gut health and have been associated with anti-inflammatory properties (Morrison and Preston, 2016). *Coprococcus* has been associated with anti-inflammatory effects and the production of butyrate (Valles-Colomer et al., 2019), which has potential anti-tumor properties. *Coriobacteriaceae*, a family of bacteria, are known to be involved in bile acid metabolism (Zhuang et al., 2021), which is critical for liver function and could play a role in carcinogenesis (Jia et al., 2018).

These microbes may influence liver cancer development through various mechanisms such as modulation of bile acids, systemic inflammation, and immune responses. Further studies are needed to elucidate the exact mechanisms through which these microbes exert their effects on liver cancer risk. Notably, the variation in the microbes associated with liver cancer risk between Europeans and East Asians suggests that genetic and environmental factors contribute to the composition of the gut microbiome and its subsequent impact on liver cancer. Diet, lifestyle, and genetic predisposition may contribute to the differing compositions of gut microbiota in these populations (Zmora et al., 2019; Beam et al., 2021), which in turn could modulate liver cancer risk through distinct pathways. This insight underscores the importance of considering population-specific factors in evaluating the role of the gut microbiome in liver cancer.

It is also essential to recognize the limitations of this study. While MR analysis inherently aids in addressing issues of confounding, it might still be susceptible to bias from pleiotropic effects where genetic variants impact multiple phenotypes. In our study, we have applied a set of sensitivity analyses such as MR-Egger and MRPRESSO methods to mitigate the possible pleiotropy. The estimates were largely consistent across the MR approaches, suggesting the robustness of our findings. Additionally, the use of summary data from GWAS necessitates caution in interpreting the results, as individual-level data could provide more nuanced insights. The cross-sectional nature of GWAS data also means that temporality cannot be established definitively. While GWAS provides robust insights into genetic associations, one inherent limitation is its cross-sectional nature, which poses challenges in conclusively establishing a temporal sequence between the microbiota changes and the onset of liver cancer (Visscher et al., 2017). Although our findings present a compelling association, it’s essential to approach them with an understanding that the directionality of this relationship is not definitively established by GWAS alone. Longitudinal studies would be more adept at ascertaining such temporality. Finally, while our study provides valuable insights into the association between gut microbiota and liver cancer, a notable limitation lies in our dataset’s inability to differentiate results based on the diverse etiologies of liver cancer such as HBV, HCV, alcohol, and NASH. This granularity is essential given that each etiology could have distinct interactions with the gut microbiota, further influenced by factors like alcohol consumption and medications for chronic hepatitis or cirrhosis. As such, the generalizability of our findings might be constrained, and readers should interpret our results within this context. Future studies with data specific to each etiology could provide a more comprehensive understanding of these interactions.

The findings of this study have several implications for future research and clinical practice. Further studies should focus on mechanistic analyses to understand the precise pathways through which the identified microbes influence liver cancer. Moreover, longitudinal studies could offer more conclusive evidence regarding causality. In clinical practice, the results highlight the potential for microbiome-targeted interventions as part of a comprehensive approach to liver cancer prevention, particularly in high-risk populations. As numerous studies have indicated, diet plays a pivotal role in shaping the gut microbiota (Beam et al., 2021). Specific dietary components, such as fibers and polyphenols, have been shown to modulate the gut microbiota in a manner that can be protective against liver carcinogenesis (Singh et al., 2018). Moreover, the administration of probiotics is another strategy that has gained attention. Probiotics, which are live beneficial bacteria, when introduced into the gut, could potentially restore or modify the gut microbiota, consequently lowering liver cancer risk (Borrelli et al., 2018).

In conclusion, our study provides novel insights into the association between the gut microbiome and liver cancer in European and East Asian populations. The identified microbes may represent potential biomarkers or therapeutic targets for liver cancer. However, further research is imperative to validate these associations and unravel the mechanisms at play. This endeavor could ultimately contribute to the development of innovative strategies for the prevention and treatment of liver cancer that are tailored to the genetic and environmental contexts of different populations.

## Data availability statement

The original contributions presented in the study are included in the article/Supplementary material, further inquiries can be directed to the corresponding authors.

## Ethics statement

This study was based on existing publicly available data. Therefore, the requirement for ethical approval and consent is waived.

## Author contributions

HJ: Conceptualization, Formal analysis, Investigation, Methodology, Software, Writing – original draft. TS: Formal analysis, Methodology, Resources, Visualization, Writing – original draft. ZL: Investigation, Supervision, Validation, Writing – review & editing. LA: Methodology, Validation, Writing – review & editing. CH: Conceptualization, Investigation, Supervision, Writing – review & editing. KZ: Conceptualization, Investigation, Supervision, Validation, Writing – review & editing.

## Conflict of interest

The authors declare that the research was conducted in the absence of any commercial or financial relationships that could be construed as a potential conflict of interest.

## Publisher’s note

All claims expressed in this article are solely those of the authors and do not necessarily represent those of their affiliated organizations, or those of the publisher, the editors and the reviewers. Any product that may be evaluated in this article, or claim that may be made by its manufacturer, is not guaranteed or endorsed by the publisher.

## Abbreviations

MR: Mendelian randomization
GWAS: Genome-wide association study
IVW: Inverse variance weighted
