# Supplementary material for: Dissecting the association between gut microbiota and liver cancer in European and East Asian populations using Mendelian randomization analysis
Source: Front Microbiol. 2023 Sep 18;14:1255650. doi: 10.3389/fmicb.2023.1255650 (PMC10544983; doi:10.3389/fmicb.2023.1255650)
Supplement: Supplementary file 1 [file Data_Sheet_1.pdf]

Supplementary materials

Dissecting the Association Between Gut Microbiota and Liver Cancer in European and East Asian Populations Using Mendelian Randomization Analysis

Table of content

Figure 1. Leave-one-out plots for the causal association between gut microbiota and liver cancer in Europeans. 2

Figure 2. Leave-one-out plots for the causal association between gut microbiota and liver cancer in East Asians. 3

Table 1. Instrumental variables for *Oscillospira*. 4

Table 2. Instrumental variables for *Mediterraneibacter gnavus group*. 5

Table 3. Instrumental variables for *Ruminococcaceae UCG010*. 6

Table 4. Instrumental variables for *Turicibacter*. 7

Table 5. Instrumental variables for *Enterobacteriaceae*. 8

Table 6. Instrumental variables for *Mollicutes RF9*. 9

Table 7. Instrumental variables for *Oscillibacter*. 10

Table 8. Instrumental variables for *Coprococcus 1*. 11

Table 9. Instrumental variables for *Coriobacteriaceae*. 12

Figure 1. Leave-one-out plots for the causal association between gut microbiota and liver cancer in Europeans.

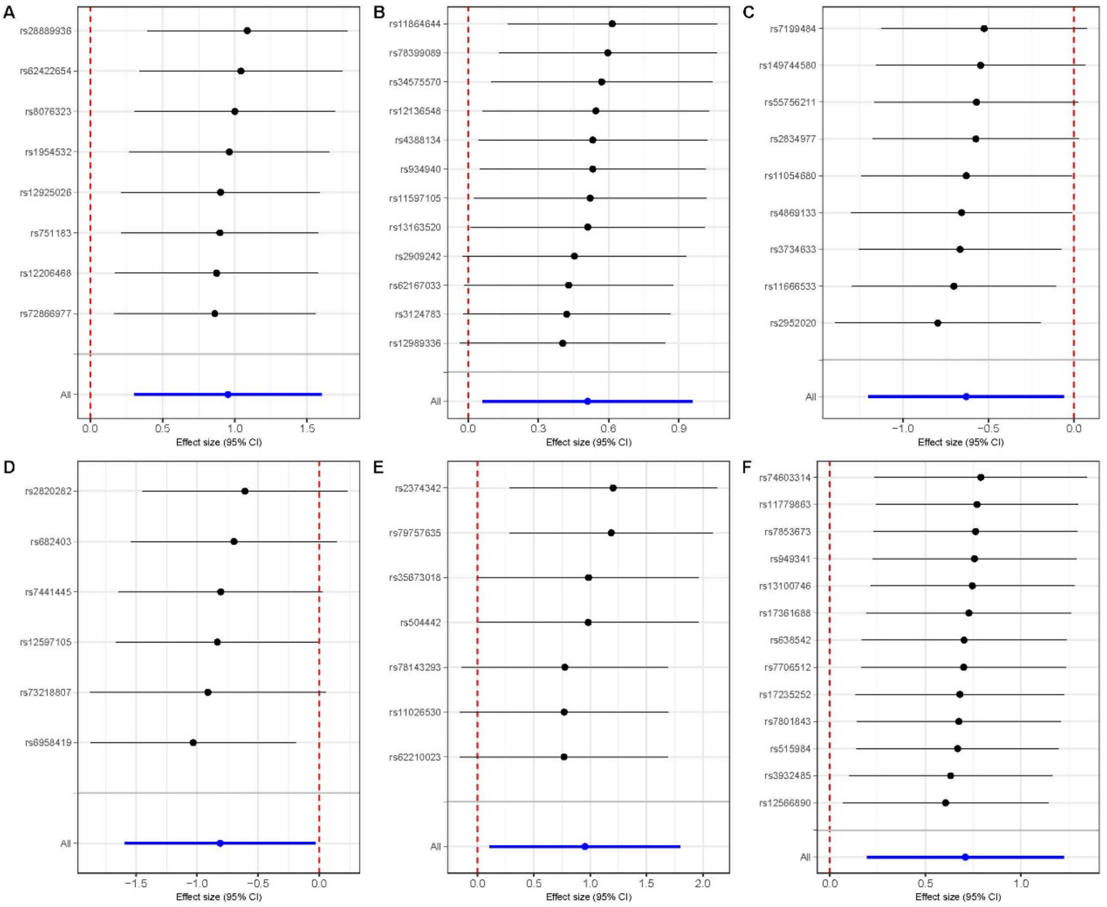

A-F denotes *Oscillospira, Mediterraneibacter gnavus group, Turicibacter, Ruminococcaceae UCG010, Enterobacteriaceae, Mollicutes RF9*, respectively.

Figure 2. Leave-one-out plots for the causal association between gut microbiota and liver cancer in East Asians.

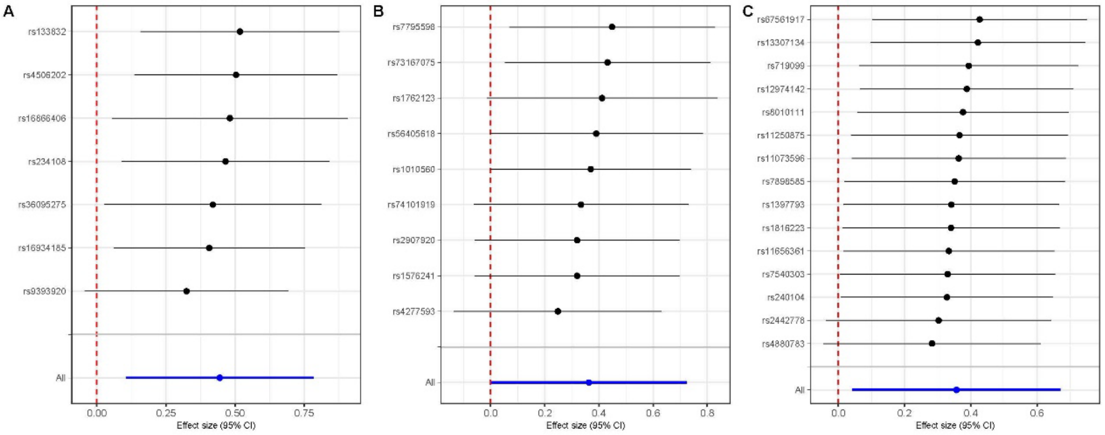

A-C denotes *Oscillbacter, Coprococcus 1,* and *Coriobacteriaceae*, respectively.

Table 1. Instrumental variables for *Oscillospira*.

SNP	effect_allele.exposure	other_allele.exposure	effect_allele.outcome	other_allele.outcome	beta.exposure	beta.outcome	chr.outcome	pos.outcome	pval.outcome	se.outcome	se.exposure	pval.exposure	mr_keep
rs12206468	G	A	G	A	-0.13302	-0.18901	6	18093460	0.103102	0.115957	0.026973	1.04E-06	TRUE
rs12925026	T	C	T	C	0.13559	0.187562	16	89726448	0.172515	0.137492	0.030678	9.31E-06	TRUE
rs1954532	T	C	T	C	-0.08262	-0.07301	14	27682209	0.352243	0.078485	0.017525	2.27E-06	TRUE
rs28889936	A	C	A	C	0.114051	0.001349	4	88562149	0.989947	0.107063	0.025284	3.37E-06	TRUE
rs62422654	C	T	C	T	0.089846	0.039936	6	1.7E+08	0.602544	0.07669	0.019809	6.47E-06	TRUE
rs72866977	A	C	A	C	-0.13057	-0.20125	6	56295685	0.087749	0.11787	0.028167	5.63E-06	TRUE
rs73038677	T	A	T	A	-0.08321	0.054609	3	1.73E+08	0.547569	0.090802	0.016867	1.09E-06	FALSE
rs751183	T	C	T	C	-0.07743	-0.11412	1	76337408	0.164154	0.082025	0.017222	6.85E-06	TRUE
rs8076323	A	G	A	G	0.071539	0.044196	17	14978543	0.509202	0.066955	0.015654	5.61E-06	TRUE

Table 2. Instrumental variables for *Mediterraneibacter gnavus group*.

SNP	effect_allele.exposure	other_allele.exposure	effect_allele.outcome	other_allele.outcome	beta.exposure	beta.outcome	chr.outcome	pos.outcome	pval.outcome	se.outcome	se.exposure	pval.exposure	mr_keep
rs11597105	A	G	A	G	0.114673	0.047652	10	6470619	0.54518	0.078764	0.025065	6.95E-06	TRUE
rs11864644	T	C	T	C	-0.1398	0.068336	16	383078	0.485671	0.098013	0.031825	5.01E-06	TRUE
rs12136548	C	T	C	T	0.090195	0.009555	1	1.15E+08	0.890201	0.069212	0.019648	3.10E-06	TRUE
rs12989336	G	A	G	A	-0.08469	-0.16594	2	36032198	0.017708	0.069966	0.018794	7.12E-06	TRUE
rs13163520	G	A	G	A	-0.12738	-0.06475	5	18662577	0.423158	0.080841	0.023388	5.61E-08	TRUE
rs2909242	C	A	C	A	-0.091	-0.10081	8	1.28E+08	0.13089	0.066735	0.018351	7.41E-07	TRUE
rs3124783	A	G	A	G	-0.11599	-0.19245	9	1.33E+08	0.03891	0.093188	0.02491	2.67E-06	TRUE
rs34575570	G	T	G	T	0.082067	-0.01568	3	66651427	0.804148	0.063234	0.017708	4.58E-06	TRUE
rs4388134	C	T	C	T	-0.0905	-0.02278	4	1.89E+08	0.745116	0.070061	0.020354	9.12E-06	TRUE
rs62167033	T	C	T	C	0.185289	0.307296	2	1.44E+08	0.048459	0.155725	0.03963	3.50E-06	TRUE
rs78399089	T	C	T	C	0.144445	-0.03867	3	1.47E+08	0.696012	0.098965	0.032662	6.63E-06	TRUE
rs934940	A	C	A	C	-0.10504	-0.01864	2	1.21E+08	0.839408	0.091971	0.022959	2.74E-06	TRUE

Table 3. Instrumental variables for *Ruminococcaceae UCG010*.

SNP	effect_allele.exposure	other_allele.exposure	effect_allele.outcome	other_allele.outcome	beta.exposure	beta.outcome	chr.outcome	pos.outcome	pval.outcome	se.outcome	se.exposure	pval.exposure	mr_keep
rs12597105	G	A	G	A	0.067085	-0.0422	16	5183940	0.595552	0.079496	0.014441	4.87E-06	TRUE
rs2820282	A	C	A	C	-0.05923	0.125681	6	1.04E+08	0.051029	0.064411	0.012592	2.85E-06	TRUE
rs35506912	G	C	G	C	-0.06937	0.058805	15	36314871	0.455581	0.078813	0.014793	3.21E-06	FALSE
rs682403	A	G	A	G	-0.05882	0.08854	9	1.33E+08	0.157272	0.062603	0.012467	2.37E-06	TRUE
rs6958419	C	T	C	T	-0.05857	-0.033	7	16310239	0.598074	0.062605	0.012499	2.84E-06	TRUE
rs73218807	G	A	G	A	-0.16621	0.103146	4	31055860	0.359254	0.112509	0.036794	6.43E-06	TRUE
rs7441445	C	T	C	T	-0.05695	0.048042	4	40705781	0.444709	0.06286	0.01265	6.80E-06	TRUE
rs7935775	A	T	A	T	-0.06314	0.011286	11	1.31E+08	0.873805	0.071055	0.013795	4.99E-06	FALSE

Table 4. Instrumental variables for *Turicibacter*.

SNP	effect_allele.exposure	other_allele.exposure	effect_allele.outcome	other_allele.outcome	beta.exposure	beta.outcome	chr.outcome	pos.outcome	pval.outcome	se.outcome	se.exposure	pval.exposure	mr_keep
rs11054680	T	C	T	C	-0.10475	0.066352	12	12089521	0.421884	0.082614	0.0227	2.31E-06	TRUE
rs11649454	G	C	G	C	0.095089	-0.12692	16	10087391	0.147616	0.087651	0.020343	3.27E-06	FALSE
rs11666533	C	T	C	T	-0.11169	-0.02149	19	11754117	0.850991	0.114396	0.024844	7.37E-06	TRUE
rs149744580	A	G	A	G	0.169883	-0.20433	2	63139827	0.140541	0.138646	0.031548	7.01E-08	TRUE
rs2221441	G	C	G	C	0.071036	-0.03947	5	1.71E+08	0.532193	0.063188	0.015343	3.46E-06	FALSE
rs2834977	T	C	T	C	-0.096	0.107214	21	35557345	0.219079	0.087238	0.020826	3.96E-06	TRUE
rs2952020	G	A	G	A	-0.0759	-0.07562	8	26170326	0.297317	0.072556	0.016576	5.63E-06	TRUE
rs3734633	G	A	G	A	-0.12096	0.014308	6	1.26E+08	0.916931	0.137184	0.02683	5.32E-06	TRUE
rs4247078	C	G	C	G	0.071038	-0.02657	15	92061868	0.677473	0.063879	0.015522	5.46E-06	FALSE
rs4869133	G	A	G	A	0.131186	-0.06935	5	96381915	0.402092	0.082771	0.027197	2.55E-06	TRUE
rs55756211	T	C	T	C	-0.11512	0.151779	7	1.31E+08	0.202139	0.118998	0.024071	2.81E-06	TRUE
rs61265175	G	C	G	C	-0.08586	-0.05806	11	1660632	0.473169	0.080941	0.018578	4.14E-06	FALSE
rs7199484	G	A	G	A	-0.07314	0.116388	16	49792388	0.087414	0.068095	0.016017	5.77E-06	TRUE
rs75286509	T	A	T	A	0.10108	0.180108	17	45078612	0.038102	0.086851	0.021634	2.83E-06	FALSE

Table 5. Instrumental variables for *Enterobacteriaceae*.

SNP	effect_allele.exposure	other_allele.exposure	effect_allele.outcome	other_allele.outcome	beta.exposure	beta.outcome	chr.outcome	pos.outcome	pval.outcome	se.outcome	se.exposure	pval.exposure	mr_keep
rs11026530	T	C	T	C	0.082241	0.158557	11	22357551	0.074073	0.08877	0.018638	9.43E-06	TRUE
rs111229068	A	T	A	T	0.110561	-0.23012	11	2542220	0.049013	0.1169	0.024172	3.65E-06	FALSE
rs2374342	C	A	C	A	0.058293	-0.02216	2	41906402	0.726733	0.0634	0.012619	4.52E-06	TRUE
rs35673018	G	A	G	A	0.089964	0.06707	16	54293833	0.536504	0.108508	0.020293	7.63E-06	TRUE
rs4792380	A	T	A	T	0.115598	-0.02252	17	13617723	0.903109	0.184971	0.025768	9.49E-06	FALSE
rs504442	T	G	T	G	0.084159	0.063701	18	57478315	0.537997	0.103437	0.018948	5.17E-06	TRUE
rs61973590	C	G	C	G	-0.06054	-0.08608	14	27248833	0.188059	0.065395	0.01337	8.54E-06	FALSE
rs62210023	A	G	A	G	0.060675	0.117544	20	56765036	0.07319	0.065607	0.013012	3.13E-06	TRUE
rs78143293	A	G	A	G	-0.08485	-0.1687	18	60005103	0.076674	0.095295	0.017042	1.20E-06	TRUE
rs79757635	C	A	C	A	0.07586	-0.05313	13	1.1E+08	0.569127	0.093313	0.017129	9.32E-06	TRUE
rs80319214	C	G	C	G	0.09904	-0.12814	2	3968343	0.290673	0.121269	0.021588	6.95E-06	FALSE

Table 6. Instrumental variables for *Mollicutes RF9*.

SNP	effect_allele.exposure	other_allele.exposure	effect_allele.outcome	other_allele.outcome	beta.exposure	beta.outcome	chr.outcome	pos.outcome	pval.outcome	se.outcome	se.exposure	pval.exposure	mr_keep
rs10071529	G	C	G	C	0.125105	0.093935	5	1.77E+08	0.353851	0.101316	0.027867	8.64E-06	FALSE
rs11779863	G	A	G	A	-0.07728	0.026015	8	15549321	0.762813	0.086203	0.017217	6.69E-06	TRUE
rs12566890	T	G	T	G	-0.10313	-0.18772	1	61385192	0.043996	0.0932	0.024177	8.11E-06	TRUE
rs13100746	C	T	C	T	0.063898	0.014138	3	1.66E+08	0.822203	0.062916	0.014269	7.29E-06	TRUE
rs17235252	T	C	T	C	-0.122	-0.11495	7	82906721	0.237065	0.09722	0.025546	2.16E-06	TRUE
rs17361688	A	G	A	G	0.08925	0.042205	3	3729786	0.623774	0.086043	0.020299	7.34E-06	TRUE
rs3932485	C	T	C	T	0.062621	0.112567	3	1.84E+08	0.07871	0.064023	0.014162	9.93E-06	TRUE
rs515984	T	C	T	C	-0.08755	-0.12369	10	3479267	0.210395	0.098756	0.019069	6.61E-06	TRUE
rs62188991	G	C	G	C	-0.11086	-0.18823	2	1.77E+08	0.160325	0.134068	0.024136	5.27E-06	FALSE
rs638542	G	A	G	A	-0.07059	-0.05576	1	1.09E+08	0.417469	0.068772	0.015705	5.17E-06	TRUE
rs739151	C	G	C	G	0.065266	0.014173	22	42345460	0.820909	0.062607	0.013977	3.09E-06	FALSE
rs74603314	T	C	T	C	0.230767	0.048647	14	46050515	0.76424	0.1622	0.04897	2.28E-06	TRUE
rs7706512	G	A	G	A	0.065735	0.053363	5	17443545	0.394167	0.062626	0.013898	2.27E-06	TRUE
rs7801843	A	G	A	G	-0.08695	-0.10102	7	4066073	0.245438	0.086974	0.019451	9.47E-06	TRUE
rs7853673	G	A	G	A	-0.06244	0.00148	9	1.14E+08	0.981174	0.062734	0.013983	6.73E-06	TRUE
rs949341	G	A	G	A	0.06566	-0.00205	11	1.13E+08	0.976668	0.070013	0.014722	7.73E-06	TRUE

Table 7. Instrumental variables for *Oscillibacter*.

SNP	effect_allele.exposure	other_allele.exposure	effect_allele.outcome	other_allele.outcome	beta.exposure	beta.outcome	chr.outcome	pos.outcome	se.outcome	pval.outcome	se.exposure	pval.exposure	mr_keep
rs12417956	C	G	C	G	0.078543	-0.00859	11	25973451	0.031405	0.78439	0.017407	6.03E-06	FALSE
rs133832	A	C	A	C	-0.07955	0.011701	22	44834707	0.04156	0.778297	0.016241	1.15E-06	TRUE
rs16866406	A	G	A	G	0.098877	0.035383	2	1.79E+08	0.031484	0.261077	0.02088	3.08E-06	TRUE
rs16934185	A	G	A	G	-0.12957	-0.18887	9	1798324	0.117849	0.109016	0.028157	4.38E-06	TRUE
rs234108	A	G	A	G	0.074955	0.018917	1	1.85E+08	0.041704	0.650126	0.015263	9.16E-07	TRUE
rs36095275	C	T	C	T	-0.07524	-0.04306	14	32270129	0.032127	0.180124	0.015686	1.40E-06	TRUE
rs4506202	A	G	A	G	-0.07113	-0.00676	8	21598077	0.032464	0.835143	0.015226	3.21E-06	TRUE
rs6901560	C	G	C	G	0.085503	-0.06949	6	36931994	0.089028	0.435097	0.018693	6.21E-06	FALSE
rs9393920	A	G	A	G	-0.07447	-0.08268	6	28580593	0.032955	0.012113	0.015108	9.92E-07	TRUE

Table 8. Instrumental variables for *Coprococcus 1*.

SNP	effect_allele.exposure	other_allele.exposure	effect_allele.outcome	other_allele.outcome	beta.exposure	beta.outcome	chr.outcome	pos.outcome	se.outcome	pval.outcome	se.exposure	pval.exposure	mr_keep
rs1010560	C	A	C	A	0.058023	-0.02327	1	30400301	0.114804	0.839347	0.012272	1.96E-06	TRUE
rs12886051	G	C	G	C	-0.05218	0.039037	14	29898456	0.039358	0.321274	0.011805	8.01E-06	FALSE
rs1576241	A	G	A	G	-0.05103	-0.04197	6	72529277	0.031906	0.188336	0.010953	3.33E-06	TRUE
rs1762123	C	T	C	T	-0.08915	-0.0196	6	1.5E+08	0.032576	0.54737	0.019855	8.01E-06	TRUE
rs2907920	A	G	A	G	0.056117	0.046005	19	2600717	0.035068	0.189562	0.012682	7.65E-06	TRUE
rs4277593	G	A	G	A	-0.05856	-0.07449	20	4319420	0.032254	0.02091	0.010991	1.14E-07	TRUE
rs56405618	A	G	A	G	-0.08963	-0.01521	4	1.74E+08	0.047053	0.746563	0.018651	1.57E-06	TRUE
rs73167075	T	C	T	C	0.057314	-0.0175	3	1.66E+08	0.034525	0.612135	0.012757	8.57E-06	TRUE
rs74101919	T	C	T	C	-0.0719	-0.03917	1	95424321	0.035663	0.272002	0.014465	1.03E-06	TRUE
rs7795598	G	A	G	A	-0.05359	0.023629	7	1.33E+08	0.031861	0.458323	0.011918	8.53E-06	TRUE

Table 9. Instrumental variables for *Coriobacteriaceae*.

SNP	effect_allele.exposure	other_allele.exposure	effect_allele.outcome	other_allele.outcome	beta.exposure	beta.outcome	chr.outcome	pos.outcome	se.outcome	pval.outcome	se.exposure	pval.exposure	mr_keep
rs11073596	T	G	T	G	0.051021	0.012151	15	86433579	0.035608	0.732916	0.011434	8.14E-06	TRUE
rs11250875	T	C	T	C	0.06075	0.015136	10	1922731	0.034531	0.661145	0.013094	4.83E-06	TRUE
rs11656361	A	C	A	C	0.077301	0.0951	17	8121332	0.077329	0.218769	0.017553	8.02E-06	TRUE
rs12974142	G	A	G	A	0.07898	-0.02581	19	52895166	0.060777	0.671072	0.01772	8.51E-06	TRUE
rs13307134	C	T	C	T	0.056569	-0.03958	7	1.05E+08	0.037726	0.294139	0.012626	7.80E-06	TRUE
rs1397793	G	A	G	A	-0.04989	-0.02957	5	90471451	0.032194	0.358382	0.011245	9.77E-06	TRUE
rs1816223	A	G	A	G	-0.05863	-0.03237	12	11494021	0.033653	0.336045	0.012901	4.84E-06	TRUE
rs240104	T	C	T	C	-0.06034	-0.07004	1	1.77E+08	0.051919	0.177313	0.012685	1.52E-06	TRUE
rs2442778	G	A	G	A	-0.11641	-0.07837	3	11654412	0.048976	0.109539	0.025855	9.03E-06	TRUE
rs4880783	G	T	G	T	0.056503	0.069092	10	1223742	0.032336	0.032622	0.012506	5.90E-06	TRUE
rs62448869	T	A	T	A	-0.04861	0.020659	7	24464799	0.034741	0.552078	0.010898	7.87E-06	FALSE
rs67561917	A	G	A	G	-0.07144	0.060332	20	62072077	0.048804	0.216379	0.015411	5.39E-06	TRUE
rs719099	A	G	A	G	0.07784	0.000894	10	65799217	0.040127	0.982229	0.015582	5.43E-07	TRUE
rs7540303	C	T	C	T	0.048323	0.037116	1	1.8E+08	0.031287	0.235497	0.010906	9.62E-06	TRUE
rs76779974	C	G	C	G	0.077489	-0.04242	5	1.25E+08	0.069188	0.539817	0.017188	5.63E-06	FALSE
rs7898585	T	C	T	C	0.07315	0.02951	10	5644995	0.036333	0.416674	0.016419	5.25E-06	TRUE
rs8010111	G	A	G	A	-0.10338	0.044251	14	39660509	0.105878	0.675988	0.022929	6.90E-06	TRUE
